# Achievements, priorities and strategies in pediatric nephrology in Europe: need for unifying approaches or acceptance of differences?

**DOI:** 10.3389/fped.2024.1458003

**Published:** 2024-12-20

**Authors:** Jochen Ehrich, Velibor Tasic, Vidar O. Edvardsson, Evgenia Preka, Larisa Prikhodina, Constantinos J. Stefanidis, Rezan Topaloglu, Diamant Shtiza, Ashot Sarkissian, Thomas Mueller-Sacherer, Rena Fataliyeva, Ina Kazyra, Elena Levtchenko, Danka Pokrajac, Dimitar Roussinov, Danko Milošević, Avraam Elia, Tomas Seeman, Mia Faerch, Inga Vainumae, Janne Kataja, Michel Tsimaratos, Irakli Rtskhiladze, Peter F. Hoyer, George Reusz, Atif Awan, Danny Lotan, Licia Peruzzi, Nazym Nigmatullina, Nasira Beishebaeva, Edite Jeruma, Augustina Jankauskiene, Olivier Niel, Valerie Said-Conti, Angela Ciuntu, Snežana Pavićević, Michiel Oosterveld, Anna Bjerre, Marcin Tkaczyk, Ana Teixeira, Adrian C. Lungu, Alexey Tsygin, Vesna Stojanović, Ludmila Podracka, Tanja Kersnik Levart, Mar Espino-Hernández, Per Brandström, Giuseppina Sparta, Harika Alpay, Dmytro Ivanov, Jan Dudley, Komiljon Khamzaev, Dieter Haffner

**Affiliations:** ^1^ Department of Pediatric Kidney, Liver, Metabolic and Neurological Diseases, Children’s Hospital, Hannover Medical School, Hannover, Germany; ^2^ Medical School, University Children’s Hospital, Skopje, North Macedonia; ^3^ Iceland Children’s Medical Center, Faculty of Medicine, School of Health Sciences, Landspitali, The National University Hospital of Iceland, University of Iceland, Reykjavik, Iceland; ^4^ Paediatric Nephrology, University Hospital Southampton NHS Foundation Trust, Southampton, United Kingdom; ^5^ Research and Clinical Institute for Pediatrics, Pirogov Russian National Research Medical University, Moscow, Russia; ^6^ Division of Pediatric Nephrology, Mitera Children’s Hospital, Athens, Greece; ^7^ Department of Pediatric Nephrology, Hacettepe University School of Medicine, Ankara, Türkiye; ^8^ Division of Pediatric Nephrology, University Hospital Centre “Mother Teresa”, Tirana, Albania; ^9^ Arabkir Joint Medical Centre Yerevan, Yerevan State Medical University, Yerevan, Armenia; ^10^ Department of Pediatric Nephrology and Gastroenterology, Medical University of Vienna, Vienna, Austria; ^11^ Division of Pediatric Nephrology, Children’s Hospital, Baku, Azerbaijan; ^12^ 1st Department of Pediatrics, Belarusian State Medical University, Minsk, Belarus; ^13^ Department of Pediatrics & Division of Pediatric Nephrology, University Hospitals Leuven, Leuven, Belgium; ^14^ Division of Pediatric Nephrology, University Children’s Hospital, Sarajevo, Bosnia and Herzegovina; ^15^ Nephrology and Hemodialysis Clinic, Department of Pediatrics, Medical University of Sofia, Sofia, Bulgaria; ^16^ Pediatric Clinic, University Hospital Center Zagreb, Zagreb, Croatia; ^17^ Division of Pediatric Nephrology, Makarios Children Hospital, Nicosia, Cyprus; ^18^ Department of Pediatrics, 2nd Medical Faculty, Charles University Prague, Prague, Czech Republic; ^19^ Department of Pediatrics and Adolescent Medicine, Aarhus University Hospital, Aarhus, Denmark; ^20^ Department of Pediatrics, University of Tartu, Tartu, Estonia; ^21^ Department of Paediatrics and Adolescents Medicine, Turku University Hospital, Turku, Finland; ^22^ Pediatric Nephrology Unit, Department of Multidisciplinary Pediatrics, Assistance Publique des Hôpitaux de Marseille, Marseille, France; ^23^ Division of Pediatric Nephrology, Medical Centre Mrcheveli, Tbilisi, Georgia; ^24^ Department of Pediatric Nephrology, University Hospital Essen, Essen, Germany; ^25^ First Department of Pediatrics, Semmelweis University, Budapest, Hungary; ^26^ Division of Pediatric Nephrology, Children’s Health Ireland (CHI) at Temple Street, Dublin, Ireland; ^27^ Division of Pediatric Nephrology, Sheba Medical Center, Edmond and Lily Children’s Hospital, Tel Hashomer, Israel; ^28^ Division of Pediatric Nephrology, Regina Margherita Children’s Hospital, Torino, Italy; ^29^ Department of Nephrology, Kazakh National Medical University, Almaty, Kazakhstan; ^30^ Division of Pediatric Nephrology, Maternity and Child Welfare National Center Under the Ministry of Health of the Kyrgyz Republic, Bishkek, Kyrgyzstan; ^31^ Division of Pediatric Nephrology, Bērnuslimībuklīnika, Nefroloģi Jasprofilavirsārste, Riga, Latvia; ^32^ Pediatric Center, Institute of Clinical Medicine, Vilnius University, Vilnius, Lithuania; ^33^ Pediatric Nephrology, Centre Hospitalier de Luxembourg, Luxembourg, Luxembourg; ^34^ Department of Child and Adolescent Health, Mater Dei Hospital, Msida, Malta; ^35^ Nephrology Unit, National Institute of Health Care for Mother and Child, Chisinau, Moldova; ^36^ Clinical Center of Montenegro, Institute for Sick Children, Podgorica, Montenegro; ^37^ Department of Paediatric Nephrology, Emma Children’s Hospital Amsterdam University Medical Center, Amsterdam, Netherlands; ^38^ Department of Pediatric and Adolescent Medicine, University Hospital of Oslo, Oslo, Norway; ^39^ Department of Pediatrics, Immunology and Nephrology, Polish Mother’s Memorial Hospital Research Institute, Lodz, Poland; ^40^ Pediatric Nephrology Division, Centro Hospitalar Universitário do Porto, Porto, Portugal; ^41^ Pediatric Nephrology, Fundeni Clincal Institute, Bucharest, Romania; ^42^ Division of Pediatric Nephrology, Institute of Pediatrics NCZD, Moscow, Russia; ^43^ Division of Pediatric Nephrology, Institute for Child and Youth Health Care of Vojvodina Novi Sad, Novi Sad, Serbia; ^44^ Department of Pediatrics, Comenius University, Bratislava, Slovakia; ^45^ Pediatric Nephrology Department, Children’s Hospital, University Medical Centre Ljubljana, Ljubljana, Slovenia; ^46^ Pediatric Nephrology, Pediatrics, University Hospital 12 Octubre, Madrid, Spain; ^47^ Pediatric Uro-Nephrologic Center, Department of Pediatrics, Queen Silvia Children’s Hospital, Sahlgrenska University Hospital, Gothenburg, Sweden; ^48^ Department of Pediatric Nephrology, University Children’s Hospital, Zurich, Switzerland; ^49^ Division of Pediatric Nephrology, Marmara University, Istanbul, Türkiye; ^50^ Nephrology and RRT Department, Shupyk National Healthcare University of Ukraine, Kyiv, Ukraine; ^51^ Division of Pediatric Nephrology, Bristol Royal Hospital for Children, Bristol, United Kingdom; ^52^ Division of Pediatric Nephrology, Pediatric Medical Institute, Tashkent, Uzbekistan

**Keywords:** European child healthcare services, nephrology, achievements, needs, workforce, prevention, rehabilitation

## Abstract

**Background:**

There is a lack of information on the current healthcare systems for children with kidney diseases across Europe. The aim of this study was to explore the different national approaches to the organization and delivery of pediatric nephrology services within Europe.

**Methods:**

In 2020, the European society for Paediatric Nephrology (ESPN) conducted a cross-sectional survey to identify the existing pediatric nephrology healthcare systems in 48 European countries covering a population of more than 200 million children.

**Results:**

The reported three most important priorities in the care of children with kidney diseases were better training of staff, more incentives for physicians to reduce staff shortages, and more hospital beds. Positive achievements in the field of pediatric nephrology included the establishment of new specialized pediatric nephrology centers, facilities for pediatric dialysis and transplant units in 18, 16, and 12 countries, respectively. The most common problems included no access to any type of dialysis (12), inadequate transplant programs for all ages of children (12), lack of well-trained physicians and dialysis nurses (12), inadequate reimbursement of hospitals for expensive therapies (10), and lack of multidisciplinary care by psychologists, dieticians, physiotherapists, social workers and vocational counsellors (6). Twenty-five of 48 countries (52%) expected to have a shortage of pediatric nephrologists in the year 2025, 63% of clinical nurses and 56% of dialysis nurses. All three groups of health care professionals were expected to be lacking in 38% of countries. Prenatal assessment and postnatal management of renal malformations by a multidisciplinary team including obstetricians, geneticists, pediatricians, and pediatric surgeons was available in one third of countries.

**Conclusions:**

Our study shows that there are still very marked differences in pediatric health care systems across the European countries and highlights the need need for appropriate services for children with kidney disease in all European countries.

## 1 Introduction

From the perspective of understanding how to improve child healthcare service systems (CHCSS), Europe's pediatric community is aware of the diversity of provision of pediatric healthcare offered in 53 different countries (1–3). However, Europe has lacked a comprehensive understanding of how this diversity affects health outcomes. Neither the pediatric workforce resources nor the training capacities and needs in pediatrics were fully understood. Differences in the delivery of pediatric nephrology care are reported for European countries since the 1990ies (4–6). However, the underlying “root-cause-effect-outcome relationships”—which are the basis of today's needs and wishes of pediatric nephrologists and their patients—are still non transparent for many countries. After the fall of the Berlin wall in 1990, general health care services changed in several East European countries from the former Soviet Union system to a Western orientated structure to fill their obvious gaps. Following the 2008 financial crisis, many East European countries started discussing changes in existing health care systems essentially as part of cost containment (7, 8). There is no information available on whether this has led to an improvement in healthcare in these countries Indeed, concern have been raised about persistent inequalities in the health status of children and adolescents with acute and chronic kidney diseases (CKD) in Europe (1, 9). This is further complicated by the gap between public health research and clinical research, and the lack of quality of statistical data on the subject (10). Compared to adults, children make up only 3% of the total CKD population and are therefore not considered a priority for a country's healthcare system (11). However, many kidney diseases and conditions in adults are inherited and manifestin early life. Using the mother and child health life course model, one would assume that investing in services for children would pay off in adulthood (12).

The European Society for Paediatric Nephrology (ESPN) is a nearly 60-year-old association aiming to strengthen the individual efforts of all European pediatric nephrologists (13). Three surveys conducted by ESPN aimed to identify the existing pediatric nephrology healthcare systems in 48 European countries covering a population of more than 200 million children (4–6). Based on the analyses of these surveys, ESPN aims to improve future services by understanding disparities and translating research into practice, with a focus on “learning across borders and making a difference”.

The first part of this article highlights the range of country profiles on national healthcare systems and policies, i.e., not only in terms of successes and failures in pediatric nephrology, but also in terms of priorities of care needs and highly specialized workforce provision in Europe in 2020. As complete and accurate official data on the logistical structures and organizational networks of pediatric nephrology centers were not available in many countries, the answers to our questions had to be based on the long-term experience of national ESPN members who are health system leaders in their countries and have consulted with their staff. The second part of this paper identifies challenges in the prenatal, preventive, rehabilitative and palliative care of children with kidney disease in order to improve the conceptualization, recommendations and standardization of multidisciplinary renal care for European children. The aim of this work is to explore the different national approaches to the organization and delivery of pediatric nephrology services and to provide a basis for comparative analysis.

## 2 Materials and methods

### 2.1 Study design

This is a cross-sectional survey designed to assess organization of European pediatric nephrology, the achievements and failures of healthcare services, needs and desires of pediatricians, workforce planning in these highly specialized centers, and multidisciplinary care in pediatric nephrology.

### 2.2 Questionnaire

A survey with twelve questions assessed the organization of renal care in children. All participants were asked to answer multiple-choice and open-ended questions. The questions about ESPN policy addressed workforce planning, health care delivery systems, organization of inpatient care for children with kidney disease, and multidisciplinary care including prenatal diagnosis, preventive treatment and rehabilitative and palliative therapy. The authors selected a leading pediatric nephrologist from each of 48 of the 53 European countries and asked them to represent their country and complete the questionnaire after consulting with colleagues where appropriate. All 48 participants were members of ESPN, either presidents of national pediatric nephrology societies or senior pediatric nephrologists in highly specialized pediatric renal centers.

### 2.3 Participating countries

Representatives from Iceland in the west to Kazakhstan in the east and from Norway in the north to Malta in the south participated in the survey. Five of 53 European countries with a total population of fewer than 200.000 inhabitants were excluded from the study. In selecting the European countries for our study, we followed the definition of Europe in the World Health Organization (WHO) list. The WHO Regional Office for Europe (WHO/Europe) is one of the six WHO regional offices in the world responsible for the WHO European Region, which comprises 53 countries.

### 2.4 Data collection and storage

The survey was administered by e-mail communication and all the 48 invited experts agreed to participate in the study. All respondents were fluent in the English language. Data were entered into the study database designed in Excel. Data completeness and accuracy assessment was conducted by JE at the coordinating site in Hanover. In the case of incomplete data, the respective survey participants were contacted and missing information collected. Part A of the survey asked for achievements and failures of national health care services for children with kidney diseases, workforce planning and ESPN policy (Table 1). Part B identifies challenges in the prenatal, preventive, rehabilitative and palliative care of children with kidney disease in order to improve the conceptualization, recommendations and standardization of multidisciplinary renal care for European children (Table 2).

**Table 1:** Questions 1.

Achievements and failures of national child health care services forchildren with kidney diseases, workforce planning and ESPN policy.
A1. Please list the top three positive achievements, unsolved problems and unsuccessful changes in renal child health care services in the last 15 years that turned out to be that had been made in your national health care system to improve childhood renal health care in children.
A2. Please list the top three priorities of CKD entities requiring urgent changes of current treatment strategies in your country.
A3. Are you expecting to have a shortage of pediatric nephrologists, ward nurses, and dialysis nurses in 2025 in your country?
A4. What institutions are involved in training young pediatric nephrologists in your country?
A5. What are the incentives for young pediatricians in your country to become pediatric nephrologists?
A6. What does your national society expect from ESPN in the near future?

**Table 2:** Questions 2.

Challenges in the prenatal, preventive, rehabilitative and palliative care of children with kidney disease in order to improve the conceptualization, recommendations and standardization of multidisciplinary renal care for European children.
B1. If prenatal counselling is desired for planning future care pathways for foetuses to detect defects of the developing kidneys and urinary tract, who is part of the counselling team for parents: obstetricians, geneticists, pediatric nephrologists, pediatric surgeons, or if other specialists, please specify where the teams work and how the cooperate?
B2. Are vaccinations administered to children with CKD by general practitioners, primary care pediatricians, pediatric nephrologists, public health facilities, or if other, please specify who administers the vaccinations?
B3. Are there analogous nephrology passports for children with kidney disease?
B4. Are electronic passports available for all children?
B5. Is rehabilitative care (including psychosocial care, schooling, health education, physiotherapy, nutritional counselling) available for children with CKD and if yes is it organized and coordinated in your country within the hospital, outside the hospital, or if elsewhere, please explain by whom and where?
B6. How is palliative care for children with long-term kidney disease organized and coordinated in your country within the hospital, outside the hospital, or if elsewhere, please explain by whom and where?

### 2.5 Statistical considerations

Data collected by the questionnaire were analyzed using descriptive statistics. When evaluating the reported data, they were not viewed as statistical facts, but as assessments and opinions of experts on the actual situation, which made statistical analyses not seen as appropriate. Therefore, similar to political opinion polls, percentages or ratios are given that could come close to the truth. For the purpose of analysis, countries were divided into groups based on (a) population size, (b) gross domestic product (GDP)/gross national product (GNP) per capita (low, lower-middle, upper-middle, and high income), (c) political systems and (d) geographic region.

## 3 Results

### 3.1 National priorities in European pediatric nephrology

Physicians from 45 countries responded to the questionnaire's open-ended question about the top three priorities in the care of long-term kidney patients that require urgent changes to current treatment strategies (Table 3). Forty-one countries each reported 1–3 priorities in relation to different needs to improve the management of services. Four countries (Croatia, Germany, Iceland and Norway) reported no need for change and 3 countries did not respond to the question. The most frequently reported priorities were better training of staff (*n* = 7), more incentives for physicians to reduce staff shortages (*n* = 3) and more hospital beds (*n* = 1), a coordinated national nephrology program for CKD patients (*n* = 1) with a focus on establishing an adequate number of high-level pediatric nephrology centers (*n* = 1), better collaboration between pediatric and adult nephrology/urology (*n* = 2), earlier referral of patients by primary care pediatricians to pediatric nephrologists (*n* = 3), and an improvement in long-term follow-up of children with CKD (*n* = 3). Furthermore, a change in legislation with approval of drugs used in adult nephrology (*n* = 1), improvement of the transplant program (*n* = 1), need for national guidelines (*n* = 1), a national registry for children with CKD (*n* = 1), telemedicine and incentives for research at university hospitals (*n* = 1) among the issues reported. Reports from 6 countries called for an improvement in the national diagnostic abilities, e.g., access to genetic testing for rare diseases (*n* = 5), improved kidney pathology services (*n* = 2), screening tests for kidney diseases (1), biomarkers for prognosis of CKD (*n* = 1) and improved criteria for diagnosis of AKI (*n* = 1). New additions to the therapeutic arsenal for the treatment of childhood kidney and urinary tract diseases was reported by 12 countries, such as the use of novel biologics and immunosuppressants for nephrotic and nephritic syndromes (*n* = 9), intensive care (*n* = 1), multidisciplinary care (*n* = 4), dietary (*n* = 1) and rehabilitative care (*n* = 1), treatment of CKD stages 2–4 (*n* = 2) and long-term follow-up for congenital kidney disease (*n* = 2).Thirteen countries specified 9 reasons explaining the need for improvement in pediatric dialysis care. Four countries called for home hemodialysis, overnight hemodialysis (*n* = 1), hemodialysis for small patients, including vascular fistulas for very young children (*n* = 1), and modern technologies (*n* = 4), catheters (*n* = 1) and biocompatible solutions (*n* = 1) for peritoneal dialysis. Ten countries reported a need for further improvement in their pediatric kidney transplant (Ktx) programs, including all types of KTx (*n* = 8), living donation (*n* = 1) and infant KTx (*n* = 1).

**Table 3:** Selection of major findings concerning priorities, successes, challenges, failures and workforce planning in European pediatric nephrology.

1. Most important priorities
– better training of staff
– more incentives for physicians to reduce staff shortages
– more hospital beds
– others
2. Positive achievements in the field of pediatric nephrology
– newly built facilities for peritoneal and hemodialysis and pediatric transplant units
– multidisciplinary care
– improved diagnostic methods
– others
3. Unsolved challenges
– no access to any type of dialysis
– inadequate transplant programs for all ages of children
– lack of well-trained physicians and dialysis nurses
– inadequate reimbursement of hospitals for expensive therapies (10)
– others
4. Unsuccessful attempts
– access to kidney transplantation
– Insufficient improvement in the fields of peritoneal or hemodialysis
– others
5. Workforce planning
– expected shortage of pediatric nephrologists, clinical nurses and dialysis nurses in the year 2025
– all three groups of health care professionals were expected to be lacking in 38% of countries.
– others

### 3.2 National successes in European pediatric nephrology

Eighteen positive achievements in the field of pediatric nephrology were reported from 46 European countries to have taken place in recent years in their national healthcare systems (Table 3). Eighteen countries had established new specialized pediatric nephrology centers. Sixteen countries had built facilities for peritoneal and hemodialysis and 12 countries had opened pediatric transplant units in the past 15 years. Accreditation of pediatric nephrology as a pediatric medical subspecialty was newly established in three countries. Multidisciplinary care became routine in 5 countries, including a new transition program to adult nephrology in one country. A standardized training program was created in one country for pediatricians. The range of diagnostic methods and abilities had expanded in 3 countries. Five countries reported improved medical and dietary care for children (2). The treatment of HUS, urinary tract infections and stones was standardized in one country. Two countries established a functioning cross-border care program to compensate for their own deficits. The diagnosis of kidney disease was improved by new techniques in six countries, and one country reported an improvement in national kidney research programs. Cost free treatment was introduced in seven countries. Treatment guidelines for doctors were published in two countries and information brochures for patients and families were published in one country.

### 3.3 National challenges in European pediatric nephrology

Forty-two countries reported up to three unresolved problems in childhood kidney care in their national health system (Table 3). The three most common problems included no access to any type of dialysis (*n* = 12), inadequate transplant programs for all ages of children (*N* = 12) and lack of well-trained physicians and dialysis nurses (*n* = 12), inadequate reimbursement of hospitals for expensive therapies (*n* = 10), lack of multidisciplinary care by psychologists, dieticians, physiotherapists, social workers and vocational counsellors (*n* = 6). The lack of (a) genetic testing (*n* = 5), (b) electronic health records systems (*n* = 2), (c) histopathology services (*n* = 2), (d) research resources (*n* = 2), (e) national registries (*n* = 1), (f) highly specialized reference centres (*n* = 2) and (g) problems of local, national and international collaboration (*n* = 1) were reported. Six countries identified communication gaps in pediatric nephrology between primary, secondary, tertiary and quaternary renal care (6), which was responsible for various problems such as overburdened outpatient clinics in tertiary and quaternary care centres, delayedor late referral of critically ill children to dialysis facilities, and also bureaucratic overload of staff members. Less frequently mentioned challenges included the drain of workforce from Eastern to Western European countries (*n* = 1), national healthcare crises (*n* = 1), high numbers of immigrants in EU countries (*n* = 1) and the lack of nationally adapted guidelines (*n* = 1). Seven countries had limited access to novel and expensive drugs, and in four countries patients had difficulty accessing highly specialized pediatric nephrology centres.

### 3.4 National failures in European pediatric nephrology

Twenty-nine countries reported that there had been unsuccessful attempts in the last 15 years to fill different gapsin childhood kidney care services (Table 3). The most frequent failure turned out to be the in access to kidney transplantation in 16 countries (*n* = 13, all from East Europe). All these countries reported that they had unsuccessfully tried to adapt transplant care in the last 15 years to the needs of children with CKD. Insufficient improvement in the fields of peritoneal or hemodialysis was reported from 7 Eastern countries. The persistent lack of pediatric nephrology centres (*n* = 2) and workforce (*n* = 7) due to insufficient training of doctors and nurses (*n* = 6), high workload (*n* = 1), or loss of specialists to other countries (*n* = 1) was reported mostly from East Europe. Managerial failures were claimed to have blocked merging between tertiary or quaternary hospitals (*n* = 3), closer cooperation between primary, secondary and tertiary care (*n* = 2) or between different pediatric nephrology centres (*n* = 3) and establishing multidisciplinary teams (*n* = 1).

### 3.5 National workforce planning in European pediatric nephrology

Regarding workforce planning, 25 of 48 countries expected to have a shortage of pediatric nephrologistsin the year 2025, 30 countries of clinical nurses and 27 of dialysis nurses (Table 3). All three groups of health care professionals were expected to be lacking in 38% of countries. A lack of pediatric nephrologists was anticipated in 14 of 28 European Union countries (EU) and in 6 of 20 Non-EU countries. The numbers were 9 of all 12 countries with high GDP/GNP per capita and 13 of 32 countries with either low or middle-income. Likewise, 9 of 10 countries with more than 21 million inhabitants reported a shortage as compared to 9 of 25 countries with a population of 4–21 million inhabitants.

The main incentives for young pediatricians to choose a training in pediatric nephrology were career opportunities in 34 of 48 countries, research in 30 and reputation in 25 and salaries in only 3 countries. Altogether 98% of countries reported that academia and research in nephrology was a key motivator for choosing pediatric nephrology, however, one third of countries reported too few pediatricians involved in research in their country. This proportion was the same for EU and Non-EU countries. The question—if there were enough qualified candidates for leading positions in highly specialized pediatric nephrology centers—was answered with “no” in 19 countries.

The national and regional planning and allocation of pediatric nephrology services in tertiary and quaternary care children's hospitals was determined by the ministries of health alone in 14 countries, together with the universities in 8 countries or by the universities alone in 6 countries and, last but not least, by the initiative of individual pioneers of pediatric nephrology in 12 countries. In the UK, the national health system was responsible for coordination of care; in the Netherlands the health insurance companies played an additional role to all of the influencers listed.

Forty-three pediatric nephrologists from 48 European countries reported that pediatric nephrology centers should be closely linked to cardiology, neonatology, intensive care and pediatric surgery/urology in highly specialized pediatric centers. Only Denmark reported a desired close contact between pediatric and adult nephrology. Pediatric nephrology was not an accredited subspecialty in one third of countries. Unfortunately, there were not enough data reported on the guidelines for accreditation of pediatric nephrology centers and for training curricula of pediatric candidates. For 27 out of 48 countries the first of the chosen top three ESPN priorities was the development of European guidelines for workforce planning in national pediatric nephrology services, secondly the development of operational manuals for nephrology service systems (*n* = 22), and thirdly written recommendations for patient pathways in outpatient renal care (*n* = 23) and multidisciplinary children's hospital care (*n* = 27).

### 3.6 Antenatal, preventive, rehabilitative and palliative care services for children with kidney disease

When congenital anomalies of the kidneys and urinary tract (CAKUT) were suspected during prenatal assessment, one third of the countries reported that obstetricians, geneticists, pediatric nephrologists and pediatric surgeons formed a joint consultation team planning postnatal care. Only in five countries did the consultation team consist of obstetricians only and in 6 countries did it consist exclusively of pediatric nephrologists. Teams of two or three specialists were reported less frequently (Table 4). Seventeen percent of countries reported the need to improve preventive care through screening and genetic testing. The need to establish a national registry of the number of patients with severe kidney disease was reported in the open questions on the most important needs of national pediatric nephrology services.

**Table 4:** Number of countries offering one to four specialists in antenatal kidney care in 48 European countries.

	Number	Number	Numbers and percentages
Type of specialist	Alone	Part of a team	Both types per 48 countries
Pediatric nephrologist	6	35	41 (87%)
Obstetrician	5	29	34 (72%)
Geneticist	0	27	27 (57%)
Surgeon	0	23	23 (49%)
Total	11	Not appl.	Not appl.
One specialist/any type	11	Not appl.	23%
Two specialists	Not appl.	10	20%
Three specialists	Not appl.	11	23%
Four specialists	Not appl.	16	34%
Total	11	37	Not appl.

In a third of countries, families were given special analogue medical passports for individual children with chronic kidney disease (CKD). Vaccinations for children with kidney disease were provided by general practitioners and different specialists. Twenty-eight countries offered a mixture of 11 different combinations of care givers. In one country the vaccines were exclusively given by pediatric nephrologists, in five countries only by general practitioners, in seven countries only by primary care pediatricians, and in seven countries only by public health facilities.

Rehabilitation, including psychosocial care, schooling, health education, physiotherapy and nutritional counselling for children with CKD, was organized and coordinated within tertiary and quaternary care children's hospitals in 15 countries and by external providers in 27 countries. Only four countries reported having special rehabilitation centers for children with kidney disease that also offer vacation dialysis. Twenty-nine percent of countries reported the need to improve rehabilitative care by supporting education and vocational training for adolescents and guiding the transition from pediatric to adult care. A quarter of countries reported the need to increase the availability of multidisciplinary teams for both inpatients and outpatients, particularly by recruiting more dieticians, psychologists and teachers. Finally, palliative care for children with severe adverse outcomes of AKI and CKD was organized and coordinated within tertiary care children's hospitals in 21 countries and through a combination of hospital and home care in 18 countries.

## 4 Discussion

Our study shows that, despite all the achievements of recent decades, there are still very significant differences in pediatric health care systems across Europe, and it highlights the need need for appropriate services for children with kidney disease in all European countries. The most common challenges included no access to any type of dialysis, the lack of kidney transplant programs for young children, well-trained physicians and dialysis nurses, adequate reimbursement of hospitals for expensive therapies, and multidisciplinary care by psychologists, dieticians, physiotherapists, social workers and vocational counsellors.

Putting the achievements and failures of the management of pediatric nephrology and their impact on health outcomes for European children with kidney diseases at the center of our survey was justified because of great diversity of healthcare and of needs and desires of pediatric nephrologists. What are the needs of young people with kidney diseases? What is the need of pediatric nephrologists for material and non-material things in a country? What is the outcome of different national strategies in pediatric nephrology? What is important, what has priority and what should politicians pay attention to? Unfortunately, the scientific literature answering these questions is scarce. The term “special healthcare” is often understood as a subjective national attitude. The late philosopher Harry Gordon Frankfurt took a different perspective on this question (14). He argued that caring for people—whether they belong to majority or minority groups—makes needs equally important. In the current paper we focussed on the various elements of competence required of pediatric nephrologists. One of the most worrying results of our survey was prospect of even fewer well-trained doctors and nurses working in the field of pediatric nephrology in the year 2025. It was therefore not surprising that one half of all reporting countries had sent an appeal to ESPN for the establishment of acollective action to develop European guidelines for workforce planning in national pediatric nephrology services, and to design operation manuals for service systems and planning pathways for renal outpatient care and multidisciplinary hospital care for kidney patients. A look at the structure of European governments showed us that interest in pediatric nephrology appears to be low in some countries. Weak points can be the fragmentation of responsibilities, which leads to a lack of uniformity, and the fact that ministries do not have a budget.

The different results concerning priorities, successes, challenges, failures and workforce planning in European pediatric nephrology cannot be discussed in detail here because of lack of published comprehensive national reports. Therefore, our article may become the basis for discussions on this issue. For instance, with respect to unsuccessful attempts, it would be interesting to know what was “managerial failure” due to regulations, leadership bias, cultural differences? Another important aspect is the role of cost-free care in 7 countries which must be explained by local experts. Moreover, several other aspects concerning roots of success, causes of failure and last but not least outcomes need to be clarified for each country.

There is a great diversity of pediatric workforce and education offered in European countries which appears to be based not so much on science but on historical factors (3). The range and quality care offered by pediatric nephrologists is endangered in those European countries reporting major deficits. In spite of an overall decrease of mortality in children under 14years of age in Europe there is a considerable concern about the fact that some countries had poorer outcomes irrespective of their Gross National Product (1). Future research should focus on the question whether this unacceptable variation could be improved by better organization of services.

Regular prenatal care matters for pregnant women. Women of childbearing age living with CKD or any type of organ transplantation should be informed on the potential risks and reported outcomes. Maternal and fetal outcomes have improved since the introduction of regular prenatal monitoring by obstetricians and nephrologists (15). Healthy pregnant women may benefit from ultrasound at certain time points to detect CAKUT (16). Pediatric nephrologists can make an important contribution to ethical decision making when they make recommendations to families about possible termination of a fetus with severe CAKUT (17). In less severe cases, they coordinate multidisciplinary postnatal management with pediatric surgeons, neonatologists, radiologists, and others (18). In our survey, one-third of European countries reported that prenatal consultation teams consist of obstetricians, geneticists, pediatric nephrologists, and pediatric surgeons. Ehrich et al. (3) reported that 42 out of 46 European countries had a medical passport for all children in which routine outpatient clinical examinations in childhood are documented. Theoretically, early documentation of kidney disease in these passports or in separate passports for children with CKD could contribute to a better long-term outcome for affected patients. However, the benefit of early detection tools such as urine sticks was less clear. Urine screening was performed in one-third of countries, and the age at screening ranged from 4 months to 6 years (19).

The current ESPN survey shows that vaccinations for children with kidney disease were provided either by family physicians, pediatricians, pediatric nephrologists, public health centers, or all of these. Half of the countries offered different combinations of vaccination centers. Immunizations of children with kidney disease are a mainstay of infection prevention. However, the individual vaccination calendar must be adapted to the specific needs and risks of kidney patients which requires the of pediatric nephrologists. Modern vaccines are generally well tolerated and permanent side effects are rare. Achieving immunity against vaccine-preventable viral and bacterial infections through early immunization prior to kidney transplantation is essential (20). Vaccination data collection and linkage to immunization information systems are integral components of this management. To this end, paper and electronic medical records should allow interoperability with these systems, including the ability to download, upload, and synchronize a child's immunization data (18).

The tradition and scope of paediatric rehabilitation in Europe varies widely, ranging from physical, sensory, intellectual, psychological and social functioning in children with CKD and disabilities (19). While some countries, such as the German-speaking countries, have largely adopted the 1980s trend of establishing pediatric rehabilitation as a separate discipline, other countries consider rehabilitation to be the responsibility of hospitals or other existing health care providers. There is still some uncertainty as to which children and adolescents with kidney disease are eligible for rehabilitation. Some legislators regarded rehabilitation as a measure to “restore the ability to work” and thus excluded children by definition. Others differentiated between congenital and acquired diseases and only provided rehabilitation for the latter (21). Whether or not children and adolescents received appropriate rehabilitation services depended largely on national regulations and, to some extent, on the individual commitment of pediatricians and other health professionals. However, rehabilitation of children with CKD and children receiving kidney replacement therapy plays a crucial role in empowering children with the association of CKD and disability and preparing young patients for adult life and social integration (22, 23). Our survey found that rehabilitative care, including psychosocial care, schooling, health education, physiotherapy and nutritional counselling, for children with CKD was mainly organized and coordinated within hospitals or in combination with multidisciplinary caregivers from outside the hospital. Very few countries reported having special rehabilitation centers for children that also offer vacation dialysis. Our previous study (2) documented “the shortage of non-physician health workers in many countries, leading to suboptimal psychosocial and nutritional support and poorly planned transition programs from pediatric to adult renal care”. Therefore, we propose the development of harmonized recommendations for the age-related rehabilitation of children with CKD according to the needs and wishes of European countries and young patients in particular.

The ideal clinical model for palliative care of young patients with advanced kidney disease is currently unknown. Internationally, outpatient renal palliative care clinics have been described with positive results (24). In our exploratory survey, we report data from the perspective of European pediatric nephrologists. We identified gaps in palliative care for children with adverse outcomes of acute and long-term kidney disease. In half of the countries, palliative care was organized and coordinated within the children's hospital or through a combination of hospital and home care. There were no reports on the role of hospices. Further studies are needed to determine the appropriate model of palliative care in pediatric nephrology (24).

A major limitation of our study is its qualitative, rather than quantitative research due to the variable availability of hard data in study centres. When planning the survey, the organisers were aware of the fact that—even if available—institutes of medical statistics did not contain enough data on pediatric nephrology; or, for political reasons, official statistics might not always reflect the true medical data in some special European countries. This mostly East European problem had been discussed by one of us (JE) with Professor Martin McKee when he was research director of the European Observatory on Health Systems and Policies. Finally, our ESPN teams had come to the conclusion in the late 1990ies that all responding national pediatric nephrologists of ESPN surveys should be very well known to ESPN. In our present survey the responders represented altogether more than a cumulative 1000 years of experience in European pediatric nephrology. Moreover, all responders knew that their individual wish was respected if confidential news should not be published or if the origin of a country should not be identifiable. Each question included the option to answer either “I don't know” or “yes or no, or other”. The percentage of “I don't know” responses to all questions given by all countries was less than 5%, indicating that the questions were well understood. When analyzing this percentage for 13 countries that were formerly part of the former USSR as republics, there were slightly more “I don't know” than indicated for the EU countries. Respondents also had the option of refusing to answer a particular question without giving a reason, but this option was very rarely used.

ESPN has taken action to close these gaps by joining forces and becoming a member of the European Kidney Health Alliance (EKHA). The EKHA is a common effort by stakeholders for the challenges of management of people with CKD in Europe through effective prevention and a more efficient care pathway. EKHA works on the principle that the issue of kidney health and disease must be considered at European level and that both the European Commission and European Parliament have vital roles to play in assisting national governments with these challenges.

## 5 Conclusions

This cross-sectional survey on the existing pediatric nephrology healthcare systems in 48 European countries showed many unmet needs. The most common problems included no access to any type of dialysis, inadequate transplant programs for all ages of children, lack of well-trained physicians and dialysis nurses, inadequate reimbursement of hospitals for expensive therapies, and lack of multidisciplinary care by psychologists, dieticians, physiotherapists, social workers and vocational counsellors. If pediatric nephrologists had too many priorities, they probably risked doing a little bit of everything, and with less success. Our study shows that there are still very marked differences in child health care systems across the European countries and that there is an urgent need to set up appropriate services for children with kidney disease in all European countries.

## Data Availability

The raw data supporting the conclusions of this article will be made available by the authors, without undue reservation.
